# Anaesthetic management and peri-anaesthetic morbidity and mortality in a population of 176 dogs undergoing mitral valve repair under cardiopulmonary bypass

**DOI:** 10.3389/fvets.2026.1800234

**Published:** 2026-03-10

**Authors:** Carolina Palacios Jimenez, Thaleia Stathopoulou, José Ignacio Redondo García, Matteo Rossanese, Daniel Brockman, Hatim Alibhai

**Affiliations:** 1 Clinical Sciences and Services, Royal Veterinary College, London, United Kingdom; 2 Departamento de Medicina y Cirugía Animal, Facultad de Veterinaria, Universidad Cardenal Herrera—CEU, Valencia, Spain

**Keywords:** anaesthesia, anaesthesia management, anaesthetic risk, cardiopulmonary bypass, dog, mitral valve

## Abstract

**Objectives:**

This study aims to describe the anaesthetic management of dogs undergoing mitral valve repair under cardiopulmonary bypass (CPB) and to evaluate the peri-anaesthetic morbidity and mortality of this procedure.

**Study design:**

This manuscript presents a retrospective, observational, descriptive cohort analysis performed at a tertiary university hospital.

**Methods:**

Medical records of dogs that had undergone mitral valve repair under CPB between August 2017 and June 2024 were examined. Demographic data, perioperative data and complications were recorded. All statistical analyses were conducted in R. The primary endpoint was all-cause mortality before discharge (dead vs. alive at discharge). Descriptive statistic and univariable and multivariable logistic regression models using Firth’s penalised maximum likelihood were fitted.

**Results:**

A total of 176 dogs underwent CPB for surgical correction of myxomatous mitral valve disease. Perioperative complications were frequent. Certain complications, particularly refractory arrhythmias and low cardiac output events, were often fatal despite aggressive intervention. The overall mortality before discharge was 20.5% (4.0% intraoperatively and 16.5% postoperatively before discharge). Lower body weight, longer CPB time and severe tricuspid regurgitation were factors associated with higher mortality.

**Conclusions and clinical relevance:**

Surgical repair of the mitral valve in dogs can be associated with serious intraoperative and postoperative complications. Careful case selection is warranted, particularly in dogs with severe tricuspid regurgitation, low body weight, or other markers of advanced disease. Where feasible, CPB duration should be minimised, and vigilant perioperative monitoring with rapid-response protocols for anticipated complications should be implemented.

## 1 Introduction

Heart disease encompasses functional, structural, or electrical abnormalities; in dogs, myxomatous mitral valve disease is the most common acquired cardiac disorder. It is estimated that approximately 10% of dogs presented to veterinary practices have heart disease. Chronic heart valvular disease is the most common heart disease of dogs in many parts of the world, and accounts for approximately 75% of canine cases of heart disease seen by veterinary practices in North America (1).

Chronic valvular disease affects mostly the mitral valve, although in approximately 30% of cases, the tricuspid valve will be affected as well (2). The disease is approximately 1.5 times more common in males than in females, and although the prevalence is higher in smaller (<20 kg) dogs, large breeds are affected as well (3).

Currently, the majority of the chronic valvular diseases are treated medically, with the aim of delaying the evolution of the pathology and the appearance of heart failure (clinical signs caused by heart dysfunction). In heart failure, either venous pressures increase so severely that fluid accumulates in the lungs or a body cavity (congestive heart failure), or the heart’s pumping ability is compromised such that it cannot meet the body’s needs either during exercise or at rest, in the face of either normal or increased venous pressures (1).

It has been shown that medical treatment can be effective and can delay the onset of heart failure for up to 15 months in dogs with myxomatous mitral valve disease compared with untreated dogs (4).

Advances in veterinary cardiac surgery and anaesthesia have enabled surgical mitral valve repair, either via minimally invasive approaches or via thoracotomy with extracorporeal circulation (5–9). Mitral valve repair surgery under cardiopulmonary bypass (CPB) is considered the most effective treatment for degenerative mitral valve disease, however minimally invasive thoracotomies might be recommended for patients on earlier stages of the disease, those seeking a less invasive approach, or when financial limitations preclude surgery.

Over the last two decades, the majority of canine mitral valve repairs under CPB have been performed at centres in Japan and the United Kingdom. Recently, other centres over the world have, or are developing Cardiothoracic Surgical programmes focused on intracardiac surgery with the use of extracorporeal circulation.

The surgical technique for these procedures has been well described (6–9). Although anaesthetists at established centres share experience and protocols with emerging programmes, detailed reporting of anaesthetic management and peri-anaesthetic complications remains limited in the veterinary literature. Therefore, the aim of this study was to describe anaesthetic management and peri-anaesthetic complications in dogs undergoing mitral valve repair under CPB, and to explore factors associated with mortality before discharge.

## 2 Materials and methods

### 2.1 Study design

This manuscript presents a retrospective, observational, descriptive cohort analysis performed at a tertiary university hospital and is reported according to the STROBE guidelines. Ethical approval was waived by the Royal Veterinary College Social Science Research Ethical Review Board because this retrospective study used fully anonymised data, with no patient follow-up and no prospective data collection. The studies were conducted in accordance with the local legislation and institutional requirements. Written informed consent was not obtained because general consent for the use of clinical data in research was obtained at the time of admission, and no additional data were collected specifically for this study. A sample size was not calculated due to the nature of the design (observational and descriptive).

### 2.2 Data collected

Medical records of dogs that had undergone mitral valve repair under CPB between August 2017 and June 2024 were examined. Data taken from the medical records included: signalment, surgery date, ACVIM (American College of Veterinary Internal Medicine) stage of disease (based on the guidelines current at the time of surgery -stage B1, B2, C or D), grade of the murmur present (I–VI), type of arrhythmia (if present), medication prior to surgery, presence and severity of pulmonary hypertension (based on echocardiography reports; mild/moderate/severe), presence and severity of tricuspid regurgitation (based on echocardiography reports; mild/moderate/severe), presence of comorbidities (osteoarthritis, epilepsy, etc), cross-clamp time (time from applying to releasing the aortic clamp, in min.), bypass time (time from connection/disconnection of CPB machine, in min.), surgery time (time from first incision to last suture, in min.), anaesthesia time (time from induction to extubation, in min.), lowest blood pressure (in mmHg) and pH during bypass, length of use of pacing wires (time from starting to stopping pacing, in min.), drugs administered intraoperatively (excluding heparin and cardioplegia), intraoperative complications (hypotension, bleeding, death, etc), survival to the end of surgery survival to discharge, cause of death (where known, or suspected cause of death), anaesthetic drugs used and DEA 1 blood type (positive or negative).

### 2.3 Standard procedures and protocols

Selection of suitable dogs, surgery planning and coordination, surgical procedure, handover to the Intensive Care Unit (ICU) team, postoperative care and debrief meeting have been described previously (10).

### 2.4 Anaesthetic protocol and management

The morning of the surgery, a physical examination of the patient was conducted. An intravenous catheter was placed on a fore limb after clipping and aseptic preparation of the area. If the patient was not amenable to catheter placement, intramuscular premedication (alfaxalone and methadone; Alfaxan Multidose, Zoetis, UK) was administered and once sedated, catheter was placed as before. If catheterisation was successful without necessitating intramuscular premedication, methadone (Comfortan, Dechra, UK) was administered intravenously (IV).

Dogs were preoxygenated for 5 min with 3–5 L/min of 100% oxygen delivered via a face mask or flow-by, depending on the tolerance of the patient. Anaesthesia was induced with propofol (PropoFlo 28, Zoetis, UK) and midazolam (Midazolam, Hameln, UK) intravenously. Trachea was intubated using a PVC cuffed endotracheal (ET) tube and the patient was connected to a rebreathing system. The endotracheal tube cuff was inflated until no leak was audible at 20 cmH_2_O. Anaesthesia was maintained at this point with isoflurane (IsoFlo, Zoetis, UK) in 100% oxygen.

When an adequate depth of anaesthesia was achieved (assessed by eye position, palpebral reflex and jaw tone), the patient was positioned in left lateral recumbency and allowed to breathe spontaneously. A multiparameter monitor (GE CareScape B650, UK) was used at this stage to monitor heart rate (HR), respiratory rate (RR), electrocardiogram (ECG), oscillometric arterial blood pressure, haemoglobin oxygen saturation (SpO_2_, %), end-tidal carbon dioxide partial pressure (EtCO_2_, mmHg), end-tidal isoflurane concentration (EtIso, %), and temperature (T, °C) every 5 min.

After clipping and aseptic preparation, a central venous catheter (3 ports) was inserted on the right jugular and a catheter was placed in a dorsal pedal artery.

The patient was then positioned in right lateral recumbency and after clipping and aseptic preparation of the thorax, a 2 point ultrasound guided thoracic paravertebral block with 0.5% of bupivacaine (MercuryPharma, UK) was performed (2 mg.kg^−1^, diluted up to 0.5 mL.kg^−1^ in total) as described by Monticelli et al. (11). The two injection points were at the level of the fifth intercostal space and the ninth intercostal space.

A urinary catheter was placed and antibiotics (cefuroxime or co-amoxiclav, each at a dose of 20 mg.kg^−1^) were administered IV before moving the patient to theatre and thereafter every 120 min.

Once in theatre, the patient was positioned in right lateral recumbency and connected to a circle breathing system. Monitoring was attached and intermittent positive pressure ventilation was started (tidal volume 10–15 mL.kg^−1^, positive end-expiratory pressure of 5 cmH_2_O and RR adjusted to maintain an EtCO_2_ of 35–40 mmHg, Mindray A7). A fentanyl (Fentadon, Dechra, UK) continuous rate infusion (CRI) was started at this time (0.1 mcg.kg.min^−1^). Anaesthesia was maintained with isoflurane (IsoFlo, Zoetis, UK) in 50% oxygen.

Atracurium (0.1 mg.kg^−1^; Hameln, UK) was administered IV and transoesophageal echocardiography (TOE) was performed. At this stage, a blood sample was taken from the jugular or the arterial catheter to assess blood gases, acid–base status, packed cell volume (PCV), total solids (TS) and activated clotting time (ACT).

Arterial cannula for the bypass circuit (via carotid artery or femoral artery) and venous drainage cannula (via right auricular appendage or left jugular vein) were inserted after the administration of sodium heparin (300 IU.kg^−1^) and checking that ACT was over 350 s.

Once connected to the bypass machine, the vaporizer was switched off and ventilation was decreased to 4 breaths per minute or totally stopped (continuous positive airway pressure of 5–7 cmH_2_O was applied on these cases). Blood pressure at this stage was managed by adjusting flow on the bypass machine and administering phenylephrine boluses as needed to maintain a mean blood pressure above 50 mmHg.

Cold (4° C) sanguinous cardioplegia solution was infused through a cannula placed in the ascending aorta following the application of an aortic cross clamp. Cardioplegia administration was repeated every 20 min or whenever electrical or mechanical cardiac activity was seen. Patient was cooled to 28° C during the surgical repair.

Arterial blood samples were taken every 30 min to check PCV, TS, acid base and electrolyte imbalances and treatment was administered if necessary (packed red cells, calcium gluconate 10% and/or sodium bicarbonate 8.4%). ACT was checked at the same time and additional doses of heparin were given if necessary.

Once the surgical repair of the valve was done and the atriotomy was ready to be closed, a dobutamine CRI (2 mcg.kg.min^−1^; Hameln, UK) was started and rewarming of the patient was initiated. As soon as the heart was closed and de-aired, warm blood cardioplegia (hot shot) was administered through the aortic cannula and the cross clamp was removed from the aorta.

Pacing wires were ready to use at this stage to support the cardiac activity, if necessary. If fibrillation occurred, electric shock, potassium chloride or cardioplegia were used to reset the heart.

Normal ventilation of the lungs was resumed once ventricular output was observed on the arterial pressure trace.

Repair of the valve was evaluated using TOE, before weaning fully off CPB. Once off, aortic and venous cannulas were removed and protamine (3 mg.kg^−1^ IV) was administered IV over 30 min. Blood boluses were given by the perfusionist through the arterial cannula at this stage to maintain the mean blood pressure between 60 and 80 mmHg.

A chest drain was placed and the thorax was closed and de-aired. Arterial cannula was removed once protamine was administered.

After protamine infusion was concluded, ACT was measured and a packed red cell transfusion was started. Once surgery was finished, the patient was positioned sternal recumbency and fentanyl CRI was decreased to 0.05 mcg.kg.min^−1^. Ultrasound examination of the thorax was performed and residual blood and air in the thorax was aspirated if needed. A recruitment manoeuvre (3 consecutive sustained inflations -continuous positive airway pressure hold for 10 s with airway pressure increased on each sustained inflation-) was applied and an arterial blood gas sample was collected to assess correct oxygenation.

Once the patient was deemed stable (adequate blood pressure and partial pressure of oxygen) and breathing spontaneously, it was transported to ICU using a portable anaesthetic trolley with all monitoring and CRIs attached.

In ICU, the patient was transferred into an oxygen kennel (80% oxygen) and extubated when swallowing was present. At this time point, the anaesthetist and the primary surgeon handed over the patient to the attending ICU clinician.

### 2.5 Statistical analysis

All analyses were conducted in R 4.5.0 (R Foundation for Statistical Computing, Vienna, Austria). The primary endpoint was all-cause mortality before discharge (dead vs. alive at discharge). Because of the modest event count and potential separation, logistic regression models using Firth’s penalised maximum likelihood, which yields bias-reduced, finite estimates under complete or quasi-complete separation were fitted. Unless otherwise noted, tests were two-sided with *α* = 0.05.

For univariable analyses, each candidate predictor was entered in a separate Firth logistic model. Continuous covariates were analysed on their native scale but reported using clinically interpretable increments: surgical times per 10 min, lowest intraoperative blood pressure per 10 mmHg, arterial pH per 0.10 units, and body weight per 1 kg. Categorical variables were coded as indicator contrasts; binary variables were analysed directly as 0/1 predictors. Given the number of preoperative and intraoperative medications assessed, *p*-values from the univariable drug models were adjusted using the Benjamini–Hochberg false discovery rate (FDR). For rare exposures or 2 × 2 tables with expected counts <5, Fisher’s exact test was used as a sensitivity check for significance; effect sizes (odds ratios, OR) and profile-likelihood 95% CIs were taken from the Firth models.

After univariable screening, a multivariable Firth logistic model to identify independent predictors was built, selecting covariates based on clinical relevance and univariable results (typically *p* < 0.10), while constraining the number of parameters relative to the number of events. To limit collinearity (particularly among time variables) variance inflation factors and pairwise correlations were assessed and avoided simultaneous inclusion of highly correlated measures. Linearity of the log-odds for continuous predictors was evaluated by visual inspection of plots of the predictor versus the modelled logit; no transformations were required in the final specification. Model inference used penalised likelihood ratio tests, and results are reported as adjusted ORs with profile-likelihood 95% CIs. Goodness-of-fit was summarised with the likelihood ratio test and by describing discrimination and calibration; no formal Hosmer-Lemeshow test was performed, given the small number of events. All multivariable analyses used complete-case data (no imputation), with the final sample size reported in the Results.

For visualisation, predicted mortality curves were generated by varying one continuous covariate across its observed range (on a clinically meaningful x-axis: minutes for times, mmHg for blood pressure, and 0.10-unit steps for pH) while holding other covariates at their sample medians (continuous) or reference levels (categorical); 95% confidence ribbons were computed from the model variance–covariance matrix.

## 3 Results

### 3.1 Demographics

A total of 176 dogs underwent CPB for surgical correction of myxomatous mitral valve disease. The median age was 10.0 years (range 2.0–15.0). The cohort was 56% male (98/176, 80 were castrated) and 44% female (78/176, 76 were spayed). Dogs were generally small breeds, with a median body weight of 6.5 kg (2.3–28.6 kg). The most common breeds were Cavalier King Charles Spaniels (39 dogs, 22%) and Chihuahuas (34 dogs, 19%). Table 1 shows the population distributed into breed categories.

**Table 1:** Distribution of breeds in a population of 176 dogs undergoing mitral valve repair under cardiopulmonary bypass.

Breed	Count out of 176
Cavalier King Charles Spaniel	39
Chihuahua	34
Australian cattle dog	1
Beagle	2
Bichon Frise	2
Border Collie	2
Border Collie X	1
Boston Terrier	2
Bruxellois Griffon	1
Cavachon	2
Cavapoo	5
Chihuahua X	11
Chinese crested	1
Cockerpoo	3
Collie	1
Coton de Tulear	1
Daschund	5
Havanese	7
Italian greyhound	1
Jack Russell Terrier	4
Jack Russell Terrier X	2
Labradoodle	5
Labrador	1
Lhasa Apso	2
Maltese	8
Maltese X	4
Mini Schnauzer	2
Norfolk Terrier	2
Pekingese	1
Pekingese X	1
Pomeranian	2
Pomeranian X	4
Poodle	2
Poodle X	2
Shih Tzu	4
Whippet	1
Yorkshire Terrier	3
Yorkshire Terrier X	2
X breed	3

X refers to cross.

### 3.2 Preoperative data

Most dogs were blood type DEA 1 positive (62%, 109/176), while the negative type was 38% (67/176). Most dogs (93%) had advanced mitral valve disease, with the majority (73%) in stage C and 20% in stage D. Only 7% were in stage B2. Almost the entire cohort (99%) was receiving pimobendan before surgery, and most were on standard heart failure medications such as spironolactone (78%), an angiotensin-converting enzyme (ACE) inhibitor (benazepril 71% or enalapril 16%), and furosemide (69%). Table 2 shows the signalment, ACVIM stage, pulmonary hypertension, tricuspid regurgitation, medication, and surgery times in the studied dogs, stratified by outcome. Table 3 shows the population distributed into weight categories.

**Table 2:** Signalment, echocardiographic findings, medication, surgery times, lowest blood pressure and lowest pH in a population of 176 dogs undergoing mitral valve repair under cardiopulmonary bypass, stratified by outcome.

Variable	Overall (*N* = 176)	ALIVE (*N* = 140)	DEAD (*N* = 36)	*p*-value^†^
Signalment				
Sex				
Male	98 (56%)	81 (58%)	17 (47%)	reference
Female	78 (44%)	59 (42%)	19 (53%)	0.254
Age (years)	10.0 (2.0–15.0)	10.0 (2.0–14.0)	10.0 (5.0–15.0)	0.640
Weight (kg)	6.5 (2.3–28.6)	7.2 (2.4–23.4)	4.6 (2.3–28.6)	0.075
Breed				
Mixed breed	25 (14%)	22 (16%)	3 (8.3%)	reference
Chihuahua and Chihuahua cross	45 (26%)	28 (20%)	17 (47%)	0.022
Cavalier King Charles Spaniel	39 (22%)	36 (26%)	3 (8.3%)	0.548
Maltese	9 (5%)	8 (5.7%)	1 (2.8%)	0.905
Other	58 (33%)	46 (33%)	12 (33%)	0.389
Blood type				
Positive	109 (61.9%)	85 (60.7%)	24 (66.7%)	reference
Negative	67 (38.1%)	55 (39.3%)	12 (33.3%)	0.532
ACVIM stage				
B2	13 (7%)	10 (7.1%)	3 (8.3%)	reference
C	128 (73%)	104 (74%)	24 (67%)	0.601
D	35 (20%)	26 (19%)	9 (25%)	0.920
Pulmonary hypertension				0.650
None	106 (60%)	86 (61%)	20 (56%)	reference
Mild	40 (23%)	30 (21%)	10 (28%)	0.395
Moderate	24 (14%)	20 (14%)	4 (11%)	0.894
Severe	6 (3%)	4 (2.9%)	2 (5.6%)	0.323
Tricuspid regurgitation				
None	57 (33%)	48 (35%)	9 (25%)	reference
Mild (vs None)	88 (50%)	70 (50%)	18 (50%)	0.550
Moderate (vs None)	24 (14%)	19 (14%)	5 (14%)	0.552
Severe (vs None)	7 (4%)	3 (2.2%)	4 (11%)	0.019
Preoperative arrhythmias				0.152
Yes	30 (17%)	21 (15%)	9 (25%)	
No	146 (83%)	119 (85%)	27 (75%)	
Preoperative medication				
Furosemide	122 (69%)	101 (72%)	21 (58%)	0.165
Pimobendan	174 (99%)	138 (99%)	36 (100%)	0.901
Benazepril	125 (71%)	102 (73%)	23 (64%)	0.327
Spironolactone	137 (78%)	107 (76%)	30 (83%)	0.488
Potassium	16 (9%)	11 (7.9%)	5 (14%)	0.314
Enalapril	28 (16%)	18 (13%)	10 (28%)	0.034
Torasemide	39 (22%)	28 (20%)	11 (31%)	0.197
Sildenafil	23 (13%)	14 (10%)	9 (25%)	0.022
Diltiazem	8 (5%)	4 (2.9%)	4 (11%)	0.045
Amlodipine	9 (5%)	6 (4.3%)	3 (8.3%)	0.396
Amiodarone	4 (2%)	3 (2.1%)	1 (2.8%)	0.902
Digoxine	3 (2%)	1 (0.7%)	2 (5.6%)	0.108
Hydrochlorothiazide	4 (2%)	3 (2.1%)	1 (2.8%)	0.902
Mexiletine	1 (0.6%)	0 (0%)	1 (2.8%)	0.208
Sacubitril with valsartan	2 (1%)	2 (1.4%)	0 (0%)	0.901
Amiloride	1 (0.6%)	0 (0%)	1 (2.8%)	0.205
Sotalol	1 (0.6%)	1 (0.7%)	0 (0%)	0.901
Surgery times				
Cross-clamp time	75.0 (43.0–119.0)	75.0 (43.0–110.0)	77.0 (53.0–119.0)	0.392
Bypass time	120.0 (81.0–237.0)	117.0 (81.0–185.0)	125.5 (95.0–237.0)	0.002
Surgery time	203.0 (135.0–360.0)	201.5 (135.0–309.0)	210.0 (164.0–360.0)	<0.001
General anaesthesia time	363.0 (259.0–757.0)	359.5 (259.0–545.0)	382.5 (281.0–757.0)	<0.001
Minutes on pacing	17.0 (0.0–1440.0)	16.0 (0.0–1440.0)	19.5 (0.0–120.0)	0.738
Physiologic variables on CPB				
Lowest BP (mmHg)	42.0 (22.0–75.0)	42.0 (25.0–75.0)	40.0 (22.0–65.0)	0.200
Lowest pH	7.3 (7.1–7.5)	7.3 (7.1–7.5)	7.3 (7.2–7.4)	0.270

Data shown as median (min–max) or n (%). ^†^*p*-values from univariate Firth logistic regression comparing DEAD vs ALIVE: for binary variables, coefficient *p*; for continuous variables, per-unit effect (the same values you modelled—no re-scaling here); for multi-level variables, individual rows show level-vs-reference *p*.

**Table 3:** Distribution of weight in a population of 176 dogs undergoing mitral valve repair under cardiopulmonary bypass.

Weight categories	Count out of 176
Less than 5 kg	53
Between 5 and 10 kg	84
Between 10.1 and 20 kg	33
More than 20 kg	6

The two more common arrhythmias noted during preoperative echocardiogram were atrial premature complexes (7%, 13/176) and atrial fibrillation (6%, 10/176). Table 4 shows the distribution of the arrhythmias in the population.

**Table 4:** Distribution of arrhythmias in a population of 176 dogs undergoing mitral valve repair under cardiopulmonary bypass.

Arrhythmia	Count out of 176
Atrial premature complexes (APCs)	12
Atrial fibrillation	10
Ventricular premature complexes (VPCs)	3
Paroxysmal atrial focal tachycardia	1
APCs and VPCs	1
Accelerated idioventricular rhythm (AIVR)	1
Supraventricular ectopic beats (SVE)	1
Supraventricular tachycardia (SVT)	2
No arrhythmia	145

60% (105/176) of the patients were not diagnosed with any other comorbidities. For the other 40% (71/176), the most popular comorbidities were osteoarthritis, dental disease, syringohydromyelia and epilepsy.

### 3.3 Perioperative data

All dogs were pre-medicated with methadone (median dose: 0.2 mg.kg^−1^ [range: 0.12–0.24]) and induced with a combination of propofol (3.12 mg.kg^−1^ [0.44–8.20]) and midazolam (0.5 mg.kg^−1^, [0.2–0.8]). Six of them received alfaxalone together with methadone for premedication (1.0 mg.kg^−1^, [0.5–1.85]). Anaesthesia was maintained with isoflurane in all the patients.

Median total anaesthesia time was approximately 6 h (median: 363 min) (range: 259–757), including a median surgical time (skin incision to closure) of 203 min (135–360). Median CPB duration was 120 min (81–237), and aortic cross-clamp time was 75 min (43–119). Non-surviving dogs tended to have longer procedure times: median CPB time was 125.5 min in non-survivors vs. 117.0 min in survivors (*p* = 0.057). Similarly, total surgery time was longer in those who died (median 210.0 min vs. 201.5 min, *p* = 0.022), and total anaesthetic time was about 23 min longer in non-survivors (median 382.5 min vs. 359.5 min, *p* = 0.016).

**Figure 1:**
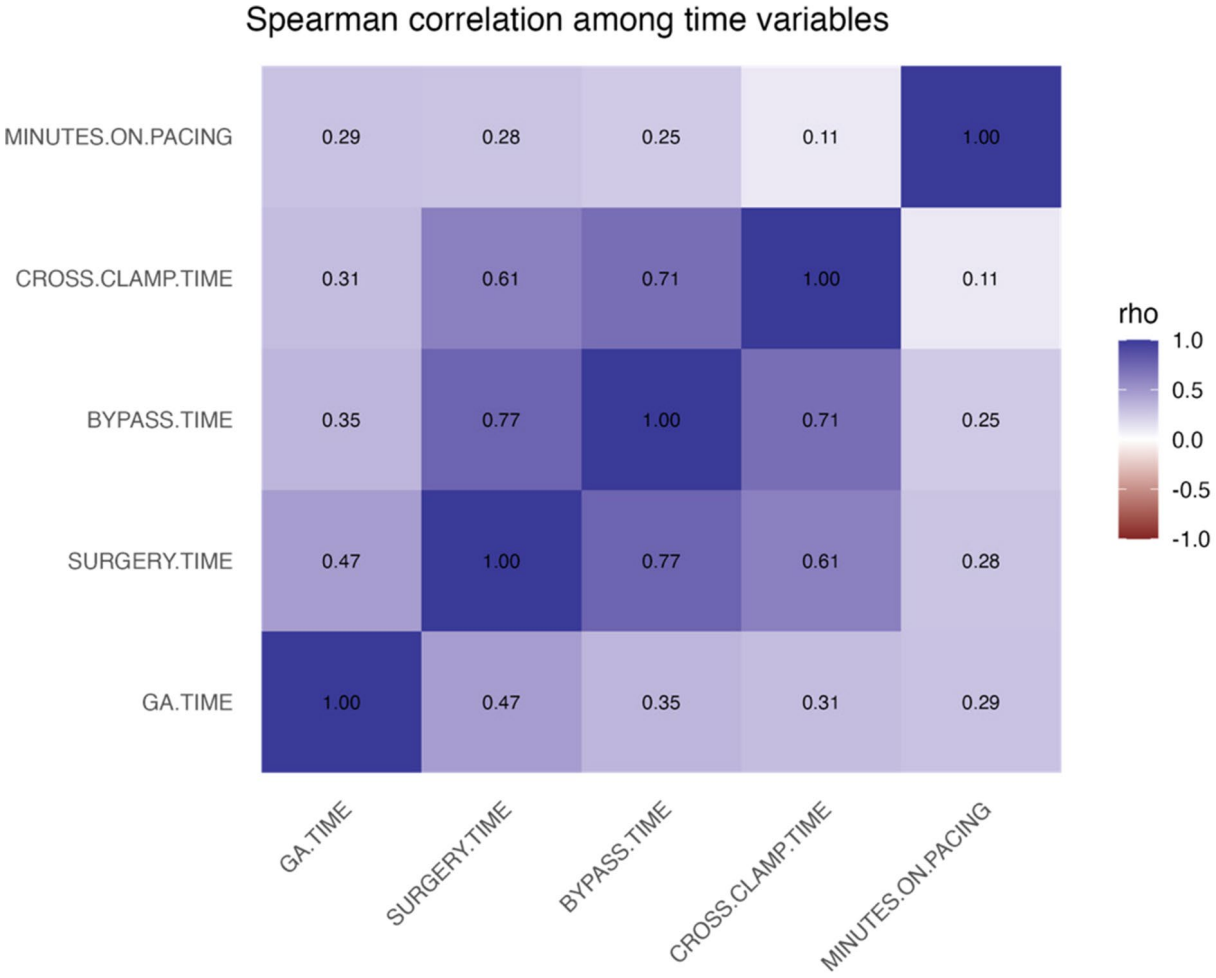
Spearman correlation heatmap among intraoperative time variables (GA time, surgery time, bypass time, cross-clamp time, minutes on pacing) in a population of 176 dogs undergoing mitral valve repair under cardiopulmonary bypass. Cells show Spearman’s ρ.

The surgical time variables were moderately to strongly correlated (Figure 1). In particular, bypass time associated with surgery time (*ρ* = 0.77) and cross-clamp time (*ρ* = 0.71), while minutes on pacing showed only weak correlations with the other durations (*ρ* ≤ 0.29).

Ventricular fibrillation occurred in 18% of the patients (31/176). Nine were treated with electrical defibrillation (Mindray BeneHeart D3 defibrillator, 0.5–1 J.kg^−1^) and twenty two were treated with potassium rich solutions. Eight of the patients that needed defibrillation did not survive to discharge.

Temporary epicardial pacing was utilised in 86% of the patients (151/176, median 20 min of pacing support, [range 1–1147]). Intraoperative haemodynamic management often required active intervention. Nearly all dogs (96%) received at least one vasoactive or adjunct drug during the bypass period. In particular, vasopressor support with phenylephrine was required in the majority of cases (82%), and 58% of dogs received sodium bicarbonate during CPB. Calcium supplementation was administered in 58% of cases. Less commonly, dogs received atropine (7%) and a small subset required epinephrine boluses (5%) or norepinephrine infusion (<3%).

### 3.4 Intra and postoperative complications

Major intraoperative complications during CPB were not frequent but clinically important. To explore whether intraoperative pharmacologic interventions were associated with perioperative complications on CPB, univariate Firth logistic models for bicarbonate, calcium, and phenylephrine (Benjamini–Hochberg FDR for multiple testing) were fitted. The results are summarised in Table 5.

**Table 5:** Intraoperative drug administration and association with perioperative complications (hypotension, hypocalcaemia and acidosis) in a population of 176 dogs undergoing mitral valve repair under cardiopulmonary bypass.

Intraoperative drugs vs. complications					
Firth logistic — OR 95% CI; BH–FDR in-between (Complication × Mode)					
	Used (*n*, %)	Not used (*n*, %)	Effect size	*p*	*p* (BH-FDR)
Global					
Bicarbonate	101 (57.4%)	75 (42.6%)	2.24 (1.19–4.34)	0.013	0.038
Calcium	103 (58.5%)	73 (41.5%)	0.61 (0.33–1.14)	0.122	0.122
Phenylephrine	140 (79.5%)	36 (20.5%)	2.18 (0.98–5.30)	0.058	0.086
Lowest. BP. during. CPB					
Bicarbonate	101 (57.4%)	75 (42.6%)	2.11 (1.10–4.19)	0.025	0.041
Calcium	103 (58.5%)	73 (41.5%)	0.54 (0.29–1.02)	0.059	0.059
Phenylephrine	140 (79.5%)	36 (20.5%)	2.62 (1.11–7.08)	0.027	0.041
Lowest. PH. during. CPB					
Bicarbonate	101 (57.4%)	75 (42.6%)	4.59 (1.56–17.93)	0.004	0.013
Calcium	103 (58.5%)	73 (41.5%)	2.35 (0.90–7.10)	0.084	0.125
Phenylephrine	140 (79.5%)	36 (20.5%)	2.21 (0.66–11.44)	0.216	0.216

Global, overall complications; Lowest. BP. during CPB, lowest blood pressure during cardiopulmonary bypass; Lowest. PH. during. CPB, lowest pH during cardiopulmonary bypass.

Results of Firth’s logistic regression models are presented as odds ratios (OR) with 95% confidence intervals.

Three percent (5/176) of patients developed haematomas in different locations of the heart and two of the patients that died experienced this complication. 5% (9/176) developed severe hypotension at some point during the anaesthetic period. Six dogs in this subset of patients developed refractory hypotension and died as a result of it (five intraoperatively and one during the postop period). 16% (28/176) experienced bleeding intraoperatively. All dogs that died intraoperatively (seven) experienced an episode of bleeding.

**Figure 2:**
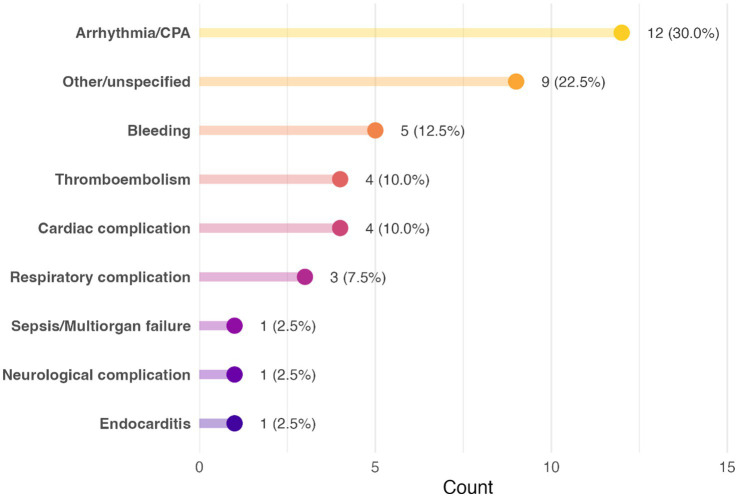
Distribution of the cause of death in deceased dogs in a population of 176 dogs undergoing mitral valve repair under cardiopulmonary bypass.

Among postoperative deaths (*n* = 29), the most common proximate cause was refractory arrhythmia or cardiopulmonary arrest in the ICU, occurring in 10 dogs (34.5% of postoperative fatalities). Other causes of postoperative death included uncontrollable haemorrhage (severe bleeding in five dogs, 17.2%), acute heart failure or low cardiac output syndrome (cardiac complications in three dogs, 10.3%), severe respiratory complications (pulmonary oedema or ventilatory failure in three dogs, 10.3%), and specific events like thromboembolism (three dogs, 10.3%), neurological events (stroke or seizure in one dog, 3.4%), endocarditis (one dog, 3.4%), and sepsis/multi-organ failure (one dog, 3.4%). Non-fatal complications were also observed in some dogs that ultimately survived to discharge. Notably, thromboembolic events (arterial thromboembolism) were recorded in four cases (2.3% of the entire cohort). Additionally, four dogs experienced significant postoperative cardiac complications (such as low-output syndrome or unplanned re-interventions, aside from arrhythmias). Three dogs had major respiratory complications (severe pulmonary dysfunction requiring prolonged ventilation) that were managed successfully. One case of confirmed endocarditis was treated medically in the postoperative period, and one dog suffered a transient neurological deficit (suspected perioperative cerebrovascular accident), from which it partially recovered. Overall, approximately 15% of the survivors experienced one or more major complications. All non-survivors encountered a catastrophic complication culminating in death, as detailed above. The distribution of proximate causes among deceased dogs is summarised in Figure 2.

Overall, 79.5% of dogs survived to discharge, whereas 20.5% died before discharge. After adjustment for multiple comparisons, no significant associations were identified between intraoperative drug administration during CPB (including vasopressors and inotropes) and the occurrence of major perioperative complications.

### 3.5 Preoperative factors and their association with mortality

The overall mortality before discharge was 20.5% (36/176). This comprised seven intraoperative deaths that occurred during CPB (4.0% of the cohort) and 29 postoperative deaths before discharge (16.5%).

Non-surviving dogs were significantly smaller in size than survivors (median weight 4.6 kg vs. 7.2 kg for survivors, *p* = 0.004). Nearly half of the dogs that died were <5 kg body weight, compared to about 20% of survivors. Breed distribution also differed by outcome: almost half of the non-survivors were Chihuahuas or Chihuahua cross (17/36, 47%), versus only 20% of survivors (28/140). Breed was not included as a variable in the multivariable model due to the limited sample size per breed.

**Figure 3:**
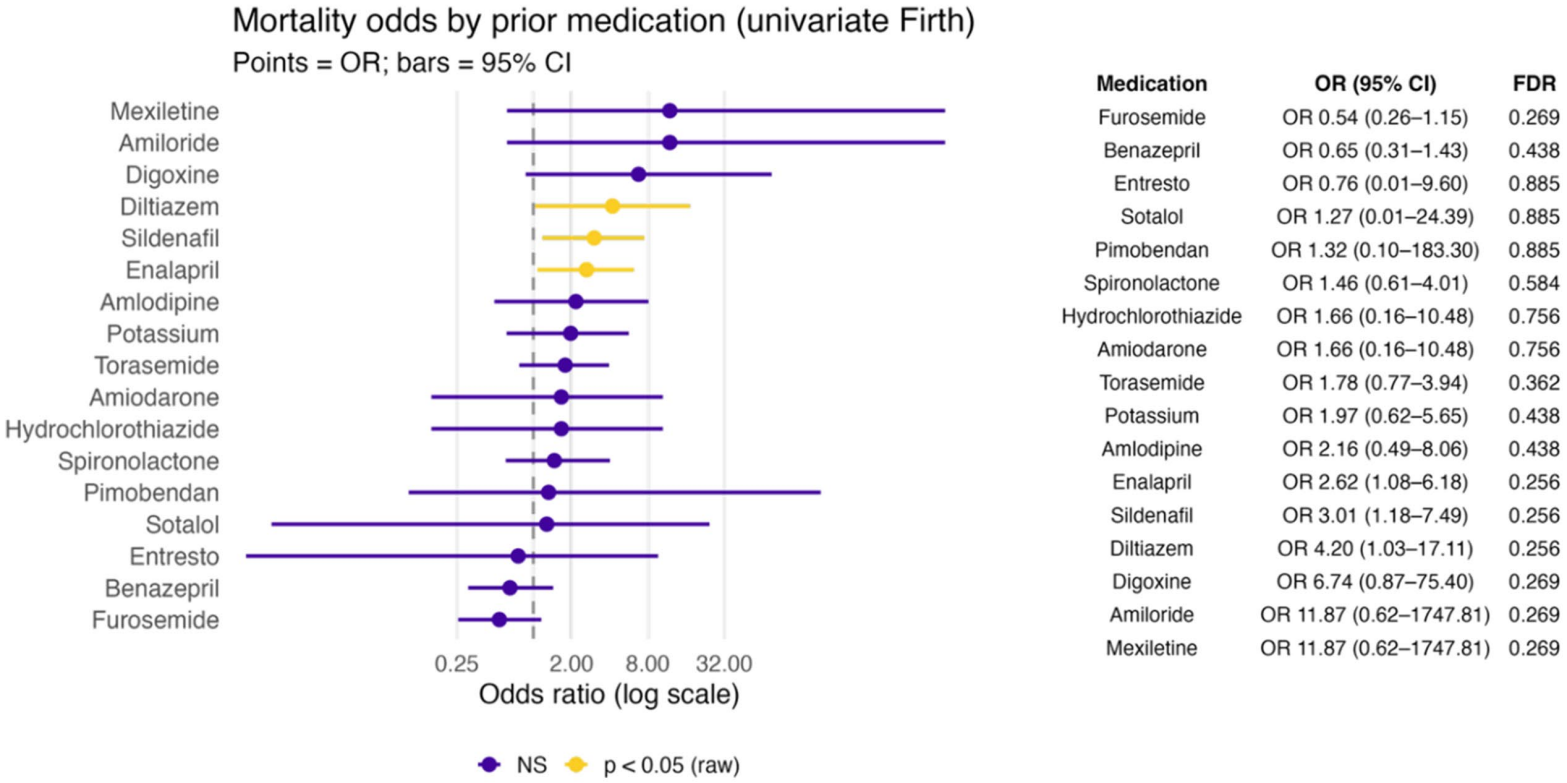
Forest plot of univariate Firth logistic regression models assessing the association between prior medication and mortality in a population of 176 dogs undergoing mitral valve repair under cardiopulmonary bypass.

None of the preoperative medications were significantly associated with survival to discharge after correcting for multiple comparisons. In univariate analyses, dogs that died had higher usage of certain drugs (25% of non-survivors had received sildenafil vs. 10% of survivors, and 28% of non-survivors were on enalapril vs. 13% of survivors). These corresponded to unadjusted odds ratios indicating roughly 3-fold higher odds of mortality with sildenafil or enalapril use (*p* = 0.026 and *p* = 0.040, respectively). However, after controlling for the false discovery rate across >15 medication comparisons, these associations were no longer statistically significant (adjusted *p* ≈ 0.32 for both). The use of furosemide was lower among dogs that died (58% vs. 72% in survivors), but this difference was not significant (*p* = 0.20). Therefore, the preoperative medication profiles did not show any clear relationship with outcome in this study after adjustment for multiple comparisons. Univariate Firth models for preoperative medications are shown in Figure 3.

### 3.6 Perioperative factors and their association with mortality

On univariate Firth logistic regression, several perioperative factors showed significant associations with mortality. Longer CPB duration was a strong predictor of death: each 10 min increase in CPB time was associated with about a 27% increase in the odds of mortality (univariate OR ≈ 1.27 per +10 min, 95% CI 1.09–1.52, *p* = 0.002). Similarly, longer total surgery time was associated with higher mortality risk (OR ≈ 1.20 per +10 min, 95% CI 1.09–1.33, *p* < 0.001), as was longer total anaesthesia time (OR ≈ 1.10 per +10 min, p < 0.001). Dogs with lower body weight faced significantly greater risk: for each 1 kg increase in weight, the odds of mortality decreased by roughly 15% (univariate OR ≈ 0.85 per +1 kg). For example, a 5 kg higher weight corresponded to an OR of about 0.44 for death, consistent with the much lower median weight observed in non-survivors. Among echocardiographic parameters, tricuspid regurgitation (TR) severity was associated with outcome in univariate analysis: dogs with severe TR had over six-fold higher odds of mortality compared to those with no TR (OR 6.43, 95% CI 1.35–33.4, *p* = 0.020). Mild or moderate TR was not significantly associated with death in univariate analysis. Other preoperative factors, such as ACVIM heart failure stage and the presence of preoperative arrhythmias, were not significant predictors of mortality on their own (*p* > 0.15 for both). Severe pulmonary hypertension was present in relatively few dogs and was not significantly associated with outcome in this sample (OR ≈ 2.3, *p* = 0.32 for severe versus none).

**Figure 4:**
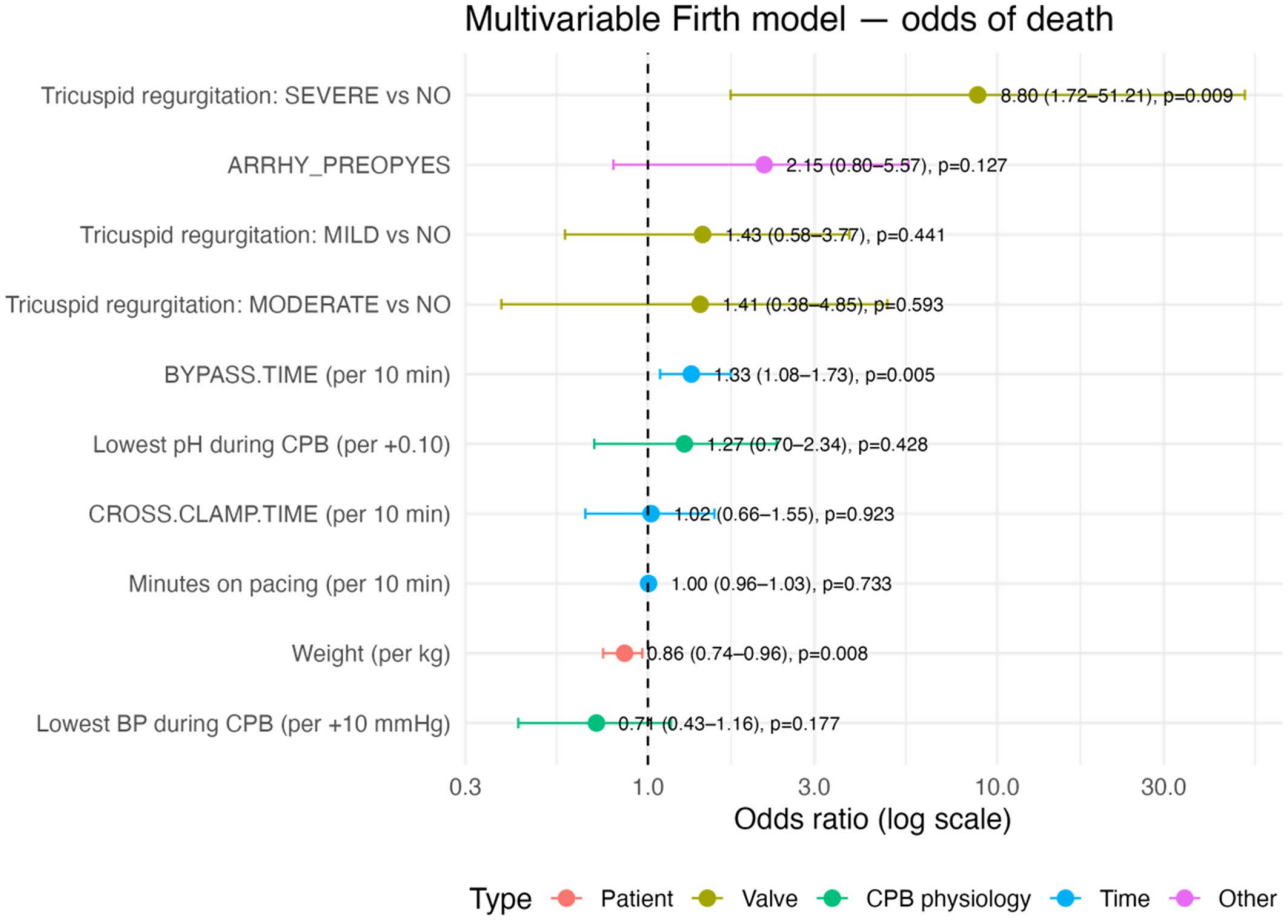
Multivariable Firth logistic regression model for predictors of death in a population of 176 dogs undergoing mitral valve repair under cardiopulmonary bypass. Forest plot of adjusted odds ratios (OR) and 95% confidence intervals for all covariates in the multivariable analysis. The plot is on a logarithmic OR scale. Dots indicate point estimates of OR, and horizontal lines represent the 95% CI. A vertical dashed line marks the line of no effect (OR = 1).

**Figure 5:**
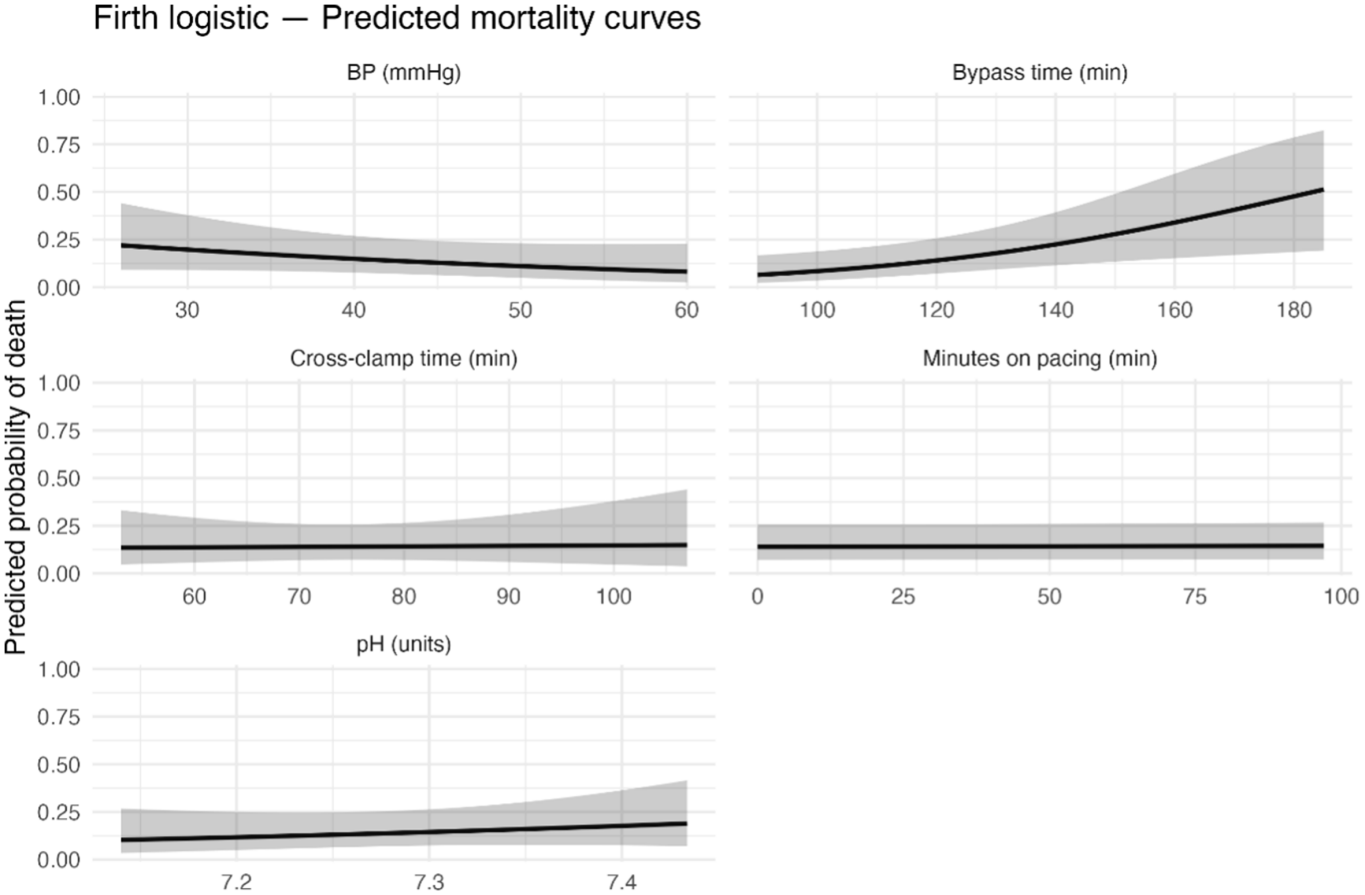
Predicted mortality curves from the multivariable Firth model in a population of 176 dogs undergoing mitral valve repair under cardiopulmonary bypass. Faceted plots illustrating the predicted probability of perioperative death according to significant clinical and intraoperative variables. Each panel displays the modeled relationship between a covariate and the estimated probability of death, holding other predictors constant. Shaded areas represent 95% confidence intervals, highlighting the uncertainty of estimates.

In constructing the multivariable model, relevant predictors from the domains above were included while considering the limited number of outcome events. The final multivariable Firth logistic regression model (Figures 4, 5) identified three independent risk factors for mortality at discharge (likelihood ratio test for the model: χ^2^ (8 df) = 24, *p* = 0.0064). Longer CPB duration remained a significant predictor of death after adjusting for other variables: each additional 10 min of bypass time increased the odds of mortality by approximately 1.33 times (adjusted OR 1.33 per + 10 min, 95% CI 1.08–1.73, *p* = 0.005). Lower body weight was independently predictive, with an adjusted OR of 0.86 per kg (95% CI 0.75–0.97, *p* = 0.008), confirming that smaller dogs had a significantly higher risk even after accounting for other factors. Severe tricuspid regurgitation was associated with markedly higher adjusted odds of mortality (OR 8.69 vs. no TR, 95% CI 1.70–50.6, *p* = 0.010). In the multivariable model, the presence of severe TR was one of the strongest predictors of death. In contrast, mild or moderate TR did not have a significant effect (adjusted OR ~1.4, *p* > 0.4 for each). Aortic cross-clamp time was also included in the model to distinguish it from overall CPB time; however, cross-clamp duration was not an independent predictor of mortality when CPB time was in the model (adjusted OR 1.02 per + 10 min, 95% CI 0.66–1.55, *p* = 0.93). Other variables in the final model (including minutes of cardiac pacing during CPB, lowest intraoperative blood pressure, lowest intraoperative pH, and preoperative arrhythmia status) did not reach statistical significance (all adjusted *p* > 0.1). For example, the odds of death tended to decrease with higher nadir blood pressure on bypass (adjusted OR ~0.71 per + 10 mmHg increase, *p* = 0.18), and the presence of a preoperative arrhythmia was associated with about twice the odds of death (OR ~2.14, *p* = 0.13). Still, neither was significant in the multivariable context. No concerning collinearity was observed among the predictors in the final model.

## 4 Discussion

This study describes anaesthetic management and peri-anaesthetic complications in dogs undergoing mitral valve repair under CPB, and reports mortality and associated risk factors. The most represented breeds were Chihuahua (or a cross Chihuahua) and the Cavalier King Charles Spaniel. The median age of these patients was 10 years and the median weight was 6.5 kg. The overall mortality before discharge was 20.5% and the main risk factors were lower body weight, longer CPB time and severe tricuspid regurgitation.

As reported before (8), serious complications arose during the intraoperative and postoperative period. The rate of these complications was high and associated with poor outcomes. This mortality rate is within the range reported for mitral valve surgery in people (2.5%–35.8%) (12). Brockman et al. (10) described a structured approach to the development of a mitral valve repair (MVR) program for dogs. Having a consistent number of cases per year, a structured, standardized approach on each case and a team of consistent personnel resulted in an improvement in outcome over time. This might reflect that improvement in both technical and non-technical skills within this complex sociotechnical environment could be the key to improving outcomes (10).

Certain complications (especially refractory arrhythmias and low cardiac output events) were often fatal despite aggressive intervention. This highlights the importance of vigilant anaesthetic management. Routine use of vasopressors, inotropes, and buffers in these cases is commonly required. Ensuring the immediate availability of drugs (phenylephrine, calcium, bicarbonate, etc.) and establishing rapid response protocols for known possible complications may facilitate timely management of recognised complications.

It has been postulated that longer cross clamp times (time in which the heart is not perfused) may potentiate postoperative cardiac dysfunction. In human patients, longer cross clamp time is associated with a higher risk of in hospital mortality, low cardiac output syndrome and acute kidney failure (13). Although Doenst et al. (13) speculated that patients with left ventricular dysfunction may have increased ischemia tolerance and therefore experience greater protection by cardioplegia than hearts with normal function. In veterinary patients, Matsuura et al. (8) reported no cases of low output syndrome or clinically relevant myocardial ischemia due to the longer arrest time. Our study showed no influence of the cross clamp time on the mortality rate.

Longer CPB duration was associated with increased odds of death. Complications frequently arise during the transition from full bypass to native circulation, when haemodynamic instability and reperfusion-related arrhythmias may occur. Prolonged CPB time may therefore reflect procedural complexity and intraoperative instability requiring ongoing support while stabilisation is achieved. In addition, extended bypass exposure can amplify inflammatory activation, contributing to systemic vasoplegia and multi-organ dysfunction. In humans (14, 15), bypass time longer than 120 min is a reliable predictor for mortality and postoperative stroke. In our study, non-survivors had a median bypass time of 125.5 min.

In humans, it is known that cardiac surgery induces a systemic inflammatory response (SIRS) mediated by CPB-triggered immune activation, myocardial ischemia–reperfusion, and direct surgical trauma (16). The concentration of the C-reactive protein (CRP) rises after major surgery, CPB or SIRS (17). In children undergoing open heart surgery, the CRP concentrations were significantly higher in those with complications than in those with uneventful recovery (17). In dogs undergoing mitral valve repair, CRP has been measured. Concentrations are not elevated immediately after surgery but they are on day 2 and 4 after surgery (18). Although an association of CRP with urinary clusterine, a marker of tubular injury, was documented in the study and could reflect a link between degree of inflammation and tubular injury, a cause–effect relationship between inflammation and acute kidney injury could not be established (18).

Ventricular fibrillation during reperfusion is a known complication during cardiac surgery in humans. The myocardium seems particularly susceptible to ventricular fibrillation right after the cross-clamp is released. At this point, fibrillation can be stopped using electrical or pharmacological defibrillation. Potassium-induced depolarization has been previously reported in human and veterinary patients (19, 20).

Ibrahim et al. (21) elucidated that blood cardioplegia significantly enhances myocardial protection by reducing arrhythmias, improving rate of recovery of function and maintaining myocardial high-energy phosphate content during ischaemia. Although later on, Guru et al. (22) showed that although blood cardioplegia provides superior myocardial protection the incidence of myocardial infarction and death are similar. Furthermore, Ovrum et al. (23), found no statistically significant differences between groups receiving cold blood or crystalloid cardioplegia concerning spontaneous sinus rhythm after aortic declamping, use of inotropic drugs, duration of ventilatory support, bleeding and rate of allogeneic blood transfusions, perioperative myocardial infarction, episodes of atrial fibrillation, stroke or minor neurological dysfunction, renal function, infections, physical rehabilitation or mortality. Even in the patients with the longest ischaemic times, they found no statistically significant differences between the groups. In veterinary patients, Kurogochi and Uechi (24), studied this topic and concluded that patients receiving blood cardioplegia had a lower incidence of fibrillation than patients receiving crystalloid cardioplegia (11.7 vs. 28.6%) and that the mortality rate did not differ between them. Therefore, they suggested that blood cardioplegia may be a superior technique for MVR in dogs. In our study, only blood cardioplegia was used and the rate of ventricular fibrillation was 18%, suggesting that despite the use of blood cardioplegia a considerable number patients developed ventricular fibrillation.

In order to try to reduce or even avoid the arrest time, there has been investigation into surgery without cross-clamping in humans (the heart is allowed to beat or fibrillate during the surgery and is perfused). Better hemodynamics and lower mortality postoperatively have been reported using this method in humans (25, 26). One veterinary report has shown the feasibility in dogs (27), although they operated in bigger dogs, therefore not applicable at the moment to the typical population that would benefit from mitral valve repair.

Cardiac haematomas, and in particular interventricular septal haematomas, are accumulations of blood that split the myocardial bundles. They can be associated with trauma, acute myocardial ischemia, or most frequently they can be a complication of cardiac surgery both in human and veterinary patients (10, 28–31).

Cardiac haematomas are easily diagnosed during TOE after valve repair or later on the postoperative period during transthoracic echocardiography. They can be life threatening as they normally cause hypomotility of the zone affected and can contribute to obstruction of the outflow tract and impaired diastolic function, which will result in hemodynamic instability. In our experience, management depends on the time of diagnosis and the haemodynamic consequences. If discovered during the surgical time, incisional drainage can be attempted. Conservative management will comprise monitoring and use of vasopressors and fluids to maintain preload and stroke volume.

Cardiac vasoplegic syndrome is a form of vasodilatory shock characterized by profound vasodilation and low systemic vascular resistance, which results in significant hypotension despite appropriate fluid resuscitation and use of vasopressors (32). Accepted definitions include SVR < 800 dynes.s.cm^−5^, MAP <60 to 65 mmHg, CI > 2.5 to 3 L.min.m^2^, and ≥1 vasopressor after adequate fluid replacement (33). The mechanism behind low SVR after cardiopulmonary bypass is poorly understood but is related to activation of an inflammatory response (34). The use of vasopressin to treat this complication has been reported in humans (35, 36). Vasopressin improved mean arterial pressure and reduced vasopressor requirements, without rebound hypertension or clinically relevant mesenteric/peripheral ischaemia.

More recently, it has been postulated that after cardiac surgery, not only the reduced plasma levels of arginine vasopressin but the excess nitric oxide (NO) production might lead to vasodilation (37). Hydroxocobalamin and methylene blue mitigate the effects of NO and both have been used successfully in human patients to treat vasoplegic syndrome refractory to catecholamines (32, 38).

Protamine sulfate is a polycationic peptide that is used in cardiothoracic surgery to reverse the anticoagulant effects of heparin. Protamine infusion is often reported to cause systemic hypotension that sometimes can lead to severe shock or even catastrophic pulmonary hypertension in susceptible individuals (39). Viaro et al. (40) reviewed the evidence for the use of methylene blue as a treatment for the distributive shock caused by protamine. This review included an *in vivo* canine study (41) that showed methylene blue and nitric oxide synthase blockers can neutralize the vasodilatory effects of protamine.

Methylene blue is well known in veterinary medicine for the treatment of methemoglobinemia or as a dye (intraoperative or in postmortem studies) but there is no clinical evidence about its use for distributive shock in veterinary patients. Nevertheless, there is some evidence from research studies (42, 43) that it could be useful treating different types of shock. At our institution, these agents are not currently stocked; their potential role in refractory vasoplegia warrants further evaluation in veterinary patients.

Bleeding was the most common intraoperative complication. It is quoted on the human literature, that up to 10% of cardiac surgical patients will experience serious bleeding and that 20% to 40% of patients will require transfusion for routine procedures. The aetiology of this bleeding is multifactorial, and includes factors like haemodilution, tissue factor activation, contact activation of the intrinsic system on circuit components, and residual effects of heparin administration. This process can lead to hyperfibrinolysis, reduced platelet count and function, decreased fibrinogen concentration, and depletion of coagulation factors culminating in impaired thrombin generation. As well, surgical trauma and the CPB circuit itself may also upregulate inflammatory cytokines, interleukins and tumour necrosis factor (44).

In human patients, the management of bleeding includes preoperative management of anticoagulants or antiplatelet agents, use of antifibrinolytics and red cell salvage, administration of pre collected autologous blood and use of different blood products (44). In veterinary patients the use of lyophilized platelet infusion, the administration of prostacyclin and the use of blood products has been described (7, 45, 46).

The use of blood products on these patients can lead to massive transfusion (transfusion of blood products of more than a blood volume over 24 h). Massive transfusion has been associated with several complications, including acute or delayed transfusion reactions, haemolysis, hypothermia, hypocalcaemia, hypomagnesemia, acidosis, and organ dysfunction (47).

In our population, bleeding was addressed either surgically (re explore of the thorax before leaving theatre or having to come back into theatre after a short stay in ICU), conservatively (with the use of blood products) or both. All patients that died intraoperatively received massive transfusions.

Recently, two papers have explored the risk predictors for early mortality after mitral valve repair in dogs (up to 100 days after surgery) (48, 49). Total bilirubin levels, TP levels, vertebral heart score, left atrial to aortic ratio and Stage D were the most predictive variables, whereas electrolytes, creatinine, peak tricuspid regurgitation velocity, fractional shortening, peak aortic jet velocity, normalized left ventricular internal diameter and Stage C were the least predictive variables.

Our endpoint was mortality before discharge, whereas previous studies evaluated mortality up to 100 days or longer. Nevertheless, our data identified severe TR, low body weight and longer CPB time as key predictors.

Dogs with severe tricuspid regurgitation preoperatively face an approximately 8- to 9-fold higher risk of perioperative mortality. Kurogochi et al. (49) identified tricuspid regurgitation velocity of >3.7 m.s^−1^ postoperatively as a risk factor for long term mortality (100–1400 days after surgery) but did not evaluate the effect on early mortality or the effect of the preoperative measures on the mortality. These patients might benefit from closer monitoring and more aggressive perioperative support. Case selection should take into account the increased risk in dogs with significant right-sided volume overload.

Lower-weight dogs had higher mortality (OR 0.87 per kg). Very small dogs may have lower cardiopulmonary reserve and can be technically more challenging to manage surgically. Tailored perioperative strategies (for example, modified perfusion approaches and closer postoperative monitoring) may therefore be warranted to optimise outcomes in this subgroup.

The requirement for medications like sildenafil or diltiazem was linked to higher mortality (although there was no statistical significance). This might indicate that dogs with severe secondary conditions (significant pulmonary hypertension or arrhythmias) could face greater surgical risks. It is recommended then to optimise these conditions before surgery and include them in the risk–benefit evaluation.

Surprisingly, being in stage C or D (heart failure) did not independently predict survival once on bypass. Instead, more specific indicators of cardiac function (such as TR severity and need for support drugs) were better at stratifying risk. This suggests that clinicians should look beyond the general heart failure stage and evaluate individual risk factors when counselling owners and planning mitral valve surgery.

Main limitation to our study was that the population comes from a single centre. Ideally, in the future, collaboration should be sought to collect data from similar centres, in order to explore the generalisability of the study results.

## 5 Conclusion

Surgical repair of the mitral valve in dogs can be associated with serious intraoperative and postoperative complications. On the basis of these findings, minimising CPB duration where feasible and implementing vigilant monitoring with rapid-response protocols for anticipated complications may help improve outcomes. During case selection and client counselling, severe tricuspid regurgitation and low body weight should be considered important risk factors for perioperative mortality.

## Data Availability

The raw data supporting the conclusions of this article will be made available by the authors, without undue reservation.

## References

[1] Keene BW Atkins CE Bonagura JD Fox PR Häggström J Fuentes VL . ACVIM consensus guidelines for the diagnosis and treatment of myxomatous mitral valve disease in dogs. J Vet Intern Med. (2019) 33:1127–40. doi: 10.1111/jvim.15488, 30974015 PMC6524084

[2] Borgarelli M Buchanan JW. Historical review, epidemiology and natural history of degenerative mitral valve disease. J Vet Cardiol. (2012) 14:93–101. doi: 10.1016/j.jvc.2012.01.011, 22386588

[3] Borgarelli M Zini E D'Agnolo G Tarducci A Santilli RA Chiavegato D . Comparison of primary mitral valve disease in German shepherd dogs and in small breeds. J Vet Cardiol. (2004) 6:27–34. doi: 10.1016/S1760-2734(06)70055-8, 19083307

[4] Boswood A Gordon SG Häggström J Wess G Stepien RL Oyama MA . Longitudinal analysis of quality of life, clinical, radiographic, echocardiographic, and laboratory variables in dogs with preclinical myxomatous mitral valve disease receiving pimobendan or placebo: the EPIC study. J Vet Intern Med. (2018) 32:72–85. doi: 10.1111/jvim.14885, 29214723 PMC5787203

[5] Petchdee S Pongkan W Lei J Jaturanratsamee K Bootcha R Meepoo W . Transcatheter edge-to-edge repair of the mitral valve in four dogs: preliminary results regarding efficacy and safety. Animals. (2024) 14:3068. doi: 10.3390/ani14213068, 39518790 PMC11545755

[6] Uechi M. Mitral valve repair in dogs. J Vet Cardiol. (2012) 14:185–92. doi: 10.1016/j.jvc.2012.01.004, 22366571

[7] Uechi M Mizukoshi T Mizuno T Mizuno M Harada K Ebisawa T . Mitral valve repair under cardiopulmonary bypass in small-breed dogs: 48 cases (2006-2009). J Am Vet Med Assoc. (2012) 240:1194–201. doi: 10.2460/javma.240.10.1194, 22559109

[8] Matsuura K Yoshida T Yamada S Aboshi Y Yotsuida H Yaginuma Y . The outcome of surgical mitral valve repair with loop-in-loop technique in dogs with different stage myxomatous mitral valve disease. J Vet Cardiol. (2022) 42:74–82. doi: 10.1016/j.jvc.2022.06.001, 35810732

[9] Isayama N Mizuno T Suzuki S Sasaki K Maeda E Uchimura Y. Surgical technique for mitral valve repair in dogs using a novel method to anchor artificial chordae tendineae with emphasis on key intraoperative decision points. Front Vet Sci. (2024) 11:1444742. doi: 10.3389/fvets.2024.1444742, 39744714 PMC11688337

[10] Brockman DJ Greensmith TD Rossanese M Young A Carey SL Boswood A . Improvement in short-term outcome over time, in a single center embarking on a canine mitral valve repair program using a structured multidisciplinary approach. Vet Surg. (2025) 54:675–85. doi: 10.1111/vsu.14229, 40018912 PMC12063714

[11] Monticelli P Jones I Viscasillas J. Ultrasound-guided thoracic paravertebral block: cadaveric study in foxes (*Vulpes vulpes*). Vet Anaesth Analg. (2017) 44:968–72. doi: 10.1016/j.vaa.2016.06.007, 28728944

[12] Abuawad H Al Shatnawi M Shawashreh R Attarakih M AlHarbi M AlShebli E . Predictors of mortality following mitral valve replacement: a systematic review. New Emirates Med J. (2024) 5:12. doi: 10.2174/0102506882312599240708105339

[13] Doenst T Berretta P Bonaros N Savini C Pitsis A Wilbring M . Aortic cross-clamp time correlates with mortality in the mini-mitral international registry. Eur J Cardiothorac Surg. (2023) 63:ezad147. doi: 10.1093/ejcts/ezad147, 37052525

[14] Bucerius J Gummert JF Borger MA Walther T Doll N Onnasch JF . Stroke after cardiac surgery: a risk factor analysis of 16,184 consecutive adult patients. Ann Thorac Surg. (2003) 75:472–8. doi: 10.1016/s0003-4975(02)04370-9, 12607656

[15] Elgariah M Omran T. Correlation between the duration of cardiopulmonary bypass time and the occurrence of morbidity and mortality in conventional adult cardiac surgery. Int J Med Arts. (2024) 4365–73. doi: 10.21608/ijma.2024.247713.1861

[16] Paparella D Yau TM Young E. Cardiopulmonary bypass induced inflammation: pathophysiology and treatment. An update. Eur J Cardiothorac Surg. (2002) 21:232–44. doi: 10.1016/s1010-7940(01)01099-5 11825729

[17] Aronen M. Value of C-reactive protein in detecting complications after open-heart surgery in children. Scand J Thorac Cardiovasc Surg. (1990) 24:141–5. doi: 10.3109/14017439009098058, 2382114

[18] Starybrat D Jepson R Bristow P Peterson S Yerramilli M Yerramilli M . Prospective evaluation of novel biomarkers of acute kidney injury in dogs following cardiac surgery under cardiopulmonary bypass. J Vet Emerg Crit Care (San Antonio). (2022) 32:733–42. doi: 10.1111/vec.13250, 36125401 PMC9826260

[19] Almdahl SM Damstuen J Eide M Mølstad P Halvorsen P Veel T. Potassium-induced conversion of ventricular fibrillation after aortic declamping. Interact Cardiovasc Thorac Surg. (2013) 16:143–50. doi: 10.1093/icvts/ivs455, 23115100 PMC3548536

[20] Kurogochi K Nii Y Chen A Uechi M. Clinical utility of pharmacological defibrillation using cardioplegic solution during canine mitral valve repair. J Vet Cardiol. (2025) 62:35–44. doi: 10.1016/j.jvc.2025.07.002, 40848537

[21] Ibrahim MF Venn GE Young CP Chambers DJ. A clinical comparative study between crystalloid and blood-based St Thomas' hospital cardioplegic solution. Eur J Cardiothorac Surg. (1999) 15:75–83. doi: 10.1016/s1010-7940(98)00287-5, 10077377

[22] Guru V Omura J Alghamdi AA Weisel R Fremes SE. Is blood superior to crystalloid cardioplegia? A meta-analysis of randomized clinical trials. Circulation. (2006) 114:I331–8. doi: 10.1161/CIRCULATIONAHA.105.001644, 16820596

[23] Øvrum E Tangen G Tølløfsrud S Øystese R Ringdal MA Istad R. Cold blood versus cold crystalloid cardioplegia: a prospective randomised study of 345 aortic valve patients. Eur J Cardiothorac Surg. (2010) 38:745–9. doi: 10.1016/j.ejcts.2010.03.052, 20452234

[24] Kurogochi K Uechi M. Blood cardioplegia reduces intraoperative ventricular fibrillation and transfusion requirements compared to crystalloid cardioplegia in canine mitral valve repair. Am J Vet Res. (2024) 85:ajvr.24.01.0017. doi: 10.2460/ajvr.24.01.0017, 38608661

[25] Katircioglu SF Cicekcioglu F Tutun U Parlar AI Babaroglu S Mungan U . On-pump beating heart mitral valve surgery without cross-clamping the aorta. J Card Surg. (2008) 23:307–11. doi: 10.1111/j.1540-8191.2008.00648.x, 18598319

[26] Wani ML Ahangar AG Singh S Irshad I Ul-Hassan N Wani SN . Efficacy and safety of beating heart mitral valve replacement. Int Cardiovasc Res J. (2014) 8:61–5. doi: 10.1016/j.athoracsur.2005.06.038, 24936483 PMC4058486

[27] Gordon-Evans WJ Carney JP Lahti MT Bianco RW. Pilot study investigating the feasibility of mitral valve repair without aortic cross-clamping and cardioplegia. Can J Vet Res. (2020) 84:159–62. 32255912 PMC7088510

[28] Vargas-Barrón J Roldán FJ Romero-Cárdenas A Molina-Carrión M Vázquez-Antona CA Zabalgoitia M . Dissecting intramyocardial hematoma: clinical presentation, pathophysiology, outcomes and delineation by echocardiography. Echocardiography. (2009) 26:254–61. doi: 10.1111/j.1540-8175.2008.00804.x, 19017318

[29] Kurosawa TA Bristow P Navarro-Cubas X Brockman D Luis FV. Interventricular septal hematoma associated with surgical mitral valve repair in four dogs. CASE (Phila). (2020) 5:43–7. doi: 10.1016/j.case.2020.11.001, 33644513 PMC7887611

[30] Savly O Soklay H Labombarda F. Interventricular septal hematoma: an unexpected complication after Perimembranous ventricular septal defect surgical repair. Echocardiography. (2025) 42:e70059. doi: 10.1111/echo.70059, 39761358

[31] Moretto L Dennler M Schreiber N Baron TM. Post-traumatic interventricular septal hematoma in a dog. J Vet Cardiol. (2025) 61:29–35. doi: 10.1016/j.jvc.2025.06.003, 40614410

[32] Peyko V Finamore M. Use of intravenous hydroxocobalamin without methylene blue for refractory Vasoplegic syndrome after cardiopulmonary bypass. Am J Case Rep. (2021) 22:e930890. doi: 10.12659/AJCR.930890, 34143764 PMC8218885

[33] Roderique JD VanDyck K Holman B Tang D Chui B Spiess BD. The use of high-dose hydroxocobalamin for vasoplegic syndrome. Ann Thorac Surg. (2014) 97:1785–6. doi: 10.1016/j.athoracsur.2013.08.050, 24792267

[34] Hill GE. Cardiopulmonary bypass-induced inflammation: is it important? J Cardiothorac Vasc Anesth. (1998) 12:21–5. 9583572

[35] Argenziano M Chen JM Choudhri AF Cullinane S Garfein E Weinberg AD . Management of vasodilatory shock after cardiac surgery: identification of predisposing factors and use of a novel pressor agent. J Thorac Cardiovasc Surg. (1998) 116:973–80. doi: 10.1016/S0022-5223(98)70049-2, 9832689

[36] Talbot MP Tremblay I Denault AY Bélisle S. Vasopressin for refractory hypotension during cardiopulmonary bypass. J Thorac Cardiovasc Surg. (2000) 120:401–2. doi: 10.1067/mtc.2000.107208, 10917960

[37] Omar S Zedan A Nugent K. Cardiac vasoplegia syndrome: pathophysiology, risk factors and treatment. Am J Med Sci. (2015) 349:80–8. doi: 10.1097/MAJ.0000000000000341, 25247756

[38] Yiu P Robin J Pattison CW. Reversal of refractory hypotension with single-dose methylene blue after coronary artery bypass surgery. J Thorac Cardiovasc Surg. (1999) 118:195–6. doi: 10.1016/S0022-5223(99)70161-3, 10384205

[39] Horrow JC. Protamine: a review of its toxicity. Anesth Analg. (1985) 64:348–61. 3883848

[40] Viaro F Dalio MB Evora PR. Catastrophic cardiovascular adverse reactions to protamine are nitric oxide/cyclic guanosine monophosphate dependent and endothelium mediated: should methylene blue be the treatment of choice? Chest. (2002) 122:1061–6. doi: 10.1378/chest.122.3.1061, 12226053

[41] Raikar GV Hisamochi K Raikar BL Schaff HV. Nitric oxide inhibition attenuates systemic hypotension produced by protamine. J Thorac Cardiovasc Surg. (1996) 111:1240–6. doi: 10.1016/s0022-5223(96)70227-1 8642826

[42] Zhang H Rogiers P Preiser JC Spapen H Manikis P Metz G . Effects of methylene blue on oxygen availability and regional blood flow during endotoxic shock. Crit Care Med. (1995) 23:1711–21. doi: 10.1097/00003246-199510000-00016, 7587237

[43] Ghiassi S Sun YS Kim VB Scott CM Nifong LW Rotondo MF . Methylene blue enhancement of resuscitation after refractory hemorrhagic shock. J Trauma. (2004) 57:515–21. doi: 10.1097/01.ta.0000136159.22721.3d 15454796

[44] Bartoszko J Karkouti K. Managing the coagulopathy associated with cardiopulmonary bypass. J Thromb Haemost. (2021) 19:617–32. doi: 10.1111/jth.15195 33251719

[45] Longmore DB. Experience with prostacyclin in cardiopulmonary bypass in dog and man. Philos Trans R Soc Lond Ser B Biol Sci. (1981) 294:18–412. doi: 10.1098/rstb.1981.0116 6117901

[46] Bode AP Lust RM Read MS Fischer TH. Correction of the bleeding time with lyophilized platelet infusions in dogs on cardiopulmonary bypass. Clin Appl Thromb Hemost. (2008) 14:38–54. doi: 10.1177/1076029607304746, 18160604

[47] Tucker C Winner A Reeves R Cooper ES Hall K Schildt J . Resuscitation patterns and massive transfusion for the critical bleeding dog-a multicentric retrospective study of 69 cases (2007-2013). Front Vet Sci. (2022) 8:788226. doi: 10.3389/fvets.2021.788226 35071385 PMC8766795

[48] Furusato S Kurogochi K Mizuno M Shinoda S Tanoshima R Uechi M. Preoperative prediction models for 30-day all-cause mortality after mitral valve repair in dogs: a single-Center retrospective cohort study. J Vet Intern Med. (2025) 39:e70152. doi: 10.1111/jvim.70152, 40451729 PMC12127052

[49] Kurogochi K Furusato S Takahashi E Tabata M Mizuno M Nii Y . Long-term outcomes of mitral valve repair with artificial chordae and annuloplasty for myxomatous mitral valve disease in dogs. J Vet Intern Med. (2025) 39:e70171. doi: 10.1111/jvim.70171 40525587 PMC12171926

